# The association between air pollution and mortality in Thailand

**DOI:** 10.1038/srep05509

**Published:** 2014-07-01

**Authors:** Yuming Guo, Shanshan Li, Benjawan Tawatsupa, Kornwipa Punnasiri, Jouni J. K. Jaakkola, Gail Williams

**Affiliations:** 1 Division of Epidemiology and Biostatistics, School of Population Health, The University of Queensland, Brisbane, Australia; 2 Shanghai Key Laboratory of Meteorology and Health, Shanghai, China; 3 Center for Environmental and Respiratory Health Research, Institute of Health Sciences, University of Oulu, Oulu, Finland; 4 Health Impact Assessment Division, Department of Heath, Ministry of Public Heath, Thailand

## Abstract

Bayesian statistical inference with a case-crossover design was used to examine the effects of air pollutants {Particulate matter <10 μm in aerodynamic diameter (PM_10_), sulphur dioxide (SO_2_), and ozone (O_3_)} on mortality. We found that all air pollutants had significant short-term impacts on non-accidental mortality. An increase of 10 μg/m^3^ in PM_10_, 10 ppb in O_3_, 1 ppb in SO_2_ were associated with a 0.40% (95% posterior interval (PI): 0.22, 0.59%), 0.78% (95% PI: 0.20, 1.35%) and 0.34% (95% PI: 0.17, 0.50%) increase of non-accidental mortality, respectively. O_3_ air pollution is significantly associated with cardiovascular mortality, while PM_10_ is significantly related to respiratory mortality. In general, the effects of all pollutants on all mortality types were higher in summer and winter than those in the rainy season. This study highlights the effects of exposure to air pollution on mortality risks in Thailand. Our findings support the Thailand government in aiming to reduce high levels of air pollution.

As Thailand transfers its economic growth from an agricultural to an industrial base^1^, it faces increasing levels of air pollution which have been found in other countries to be associated with adverse health consequences. Particulate matter <10 μm in aerodynamic diameter (PM_10_) is the most important air pollutant in both urban and rural areas, the concentration of which is as high as or higher than those in North America and European countries^2^. Meanwhile, Thailand is also experiencing high ozone (O_3_) pollution because of the intense emission of air pollution coupled with year-round sunlight^3^. Even in the rural areas, forest fires and agricultural burning have contributed to high levels of air pollution, which have increased to critical levels since 2006^1^,^4^. However, in order to continue the economic growth, expansion of petrochemical plants and industries rose sharply, which greatly contribute to air pollution.

Many studies worldwide have shown that short-term exposure to air pollution is associated with mortality and morbidity including cardiovascular and respiratory diseases, exacerbation of chronic respiratory conditions, and decreased lung function^5^,^6^,^7^,^8^,^9^. However, only a few studies on air pollution and mortality have been conducted in Thailand^2^,^10^. Data were limited to Bangkok, where the estimated effect of air pollution on mortality was higher than Hong Kong, Shanghai and Wuhan^10^. Measurements of air pollution-health associations are limited in other areas in Thailand. In addition, single-city studies have limited generalisability and it is difficult to combine their estimates of air pollution effects, because different modelling approaches have been used. Therefore, it is necessary and important to conduct a multi-city analysis of air pollution in Thailand, where the characteristics of air pollution (e.g., level and components) and socio-demographic status of local residents (e.g., disease pattern, and socioeconomic status) are different from those of developed countries.

In this study, we examined the association between daily air pollutants (PM_10_, O_3_, and sulphur dioxide (SO_2_)) and mortality (non-accidental, cardiovascular and respiratory diseases) in 18 Thailand provinces (Figure 1). We also examined whether the associations differed by season.

## Results

**Figure 1:**
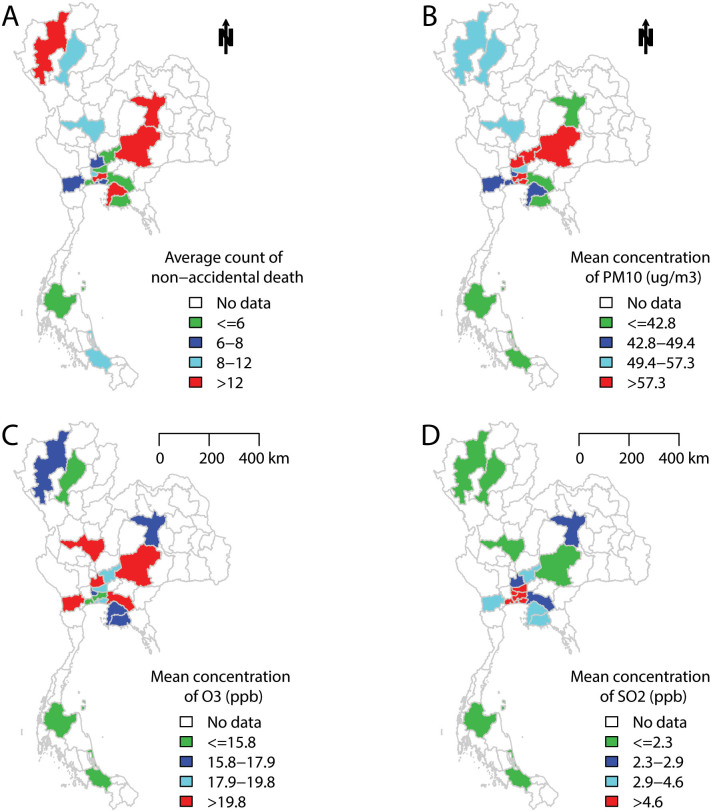
The mean values of (A) non-accidental death, (B) PM_10_, (C) O_3_, and (D) SO_2_ in 18 Thailand provinces during 1999–2008. R software with “maptools package” was used to produce the maps.

Table 1 summarises mortality, air pollution, temperature and relative humidity data in the 18 Thailand provinces. Daily average counts of non-accidental, cardiovascular, and respiratory deaths differed by province and ranged from 4 to 66, from 1 to 14, and from 1 to 8, respectively. Daily mean concentrations of PM_10_, O_3_ and SO_2_ in the Thailand provinces ranged from 31.2 to 94.9 μg/m^3^, from 9.9 to 25.6 ppb, and from 1.1 to 13.9 ppb, respectively. All provinces had similar temperature and relative humidity. In general, the air pollution levels are higher in north middle provinces than southern Thailand (Figure 1). Similar pattern was found for non-accidental deaths.

Table 2 shows the mean values for the Spearman correlation coefficients between air pollutants and weather conditions in 18 provinces in Thailand. PM_10_, O_3_ and SO_2_ were positively correlated with each other, except that O_3_ and SO_2_ had slightly positive correlation. Temperature and relative humidity were negatively correlated with PM_10_ and O_3_. The results for each province are shown in supplemental material Table S1.

**Figure 2:**
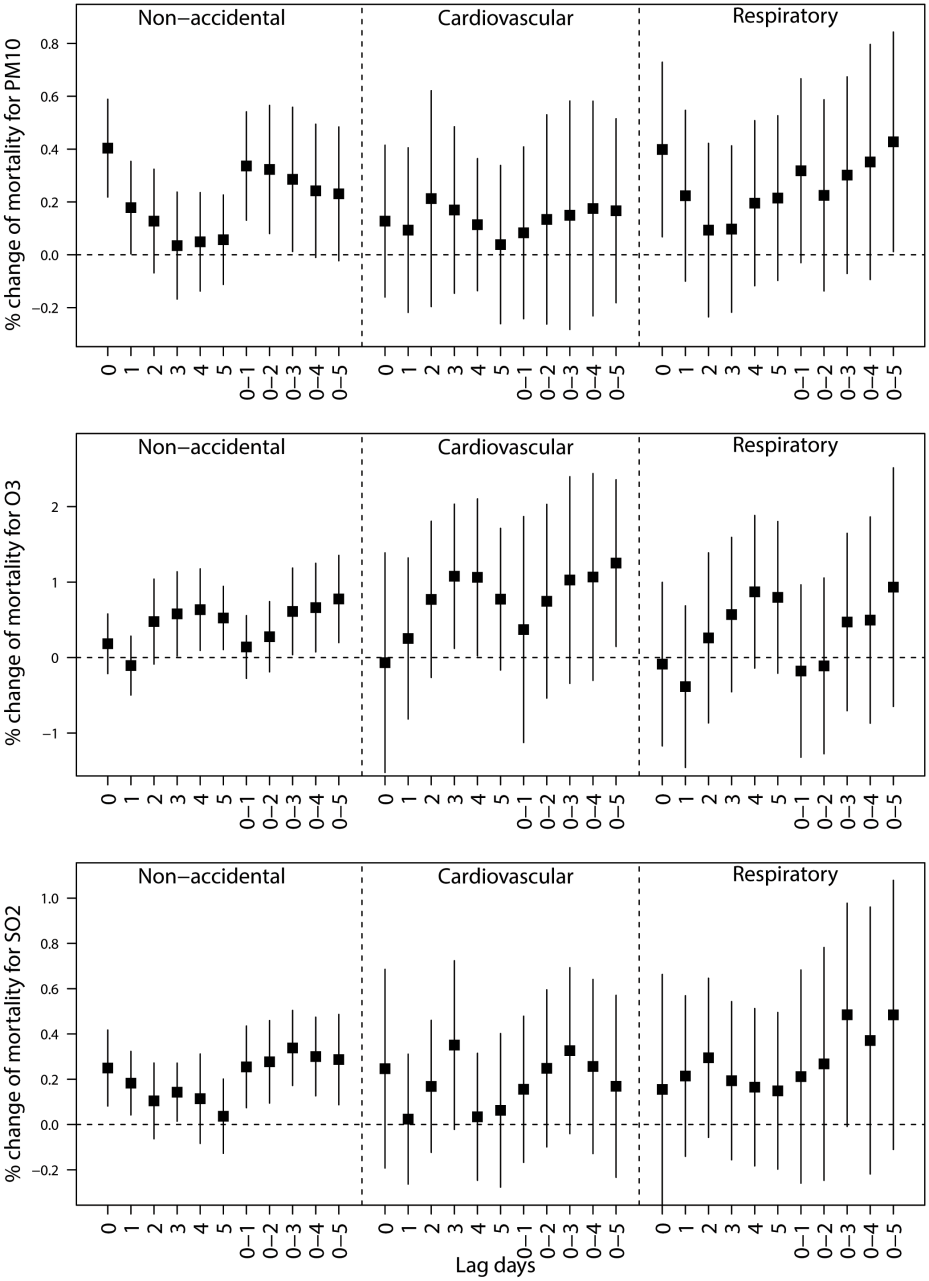
Percentage change (mean and 95% PIs) of mortality associated with a 10 μg/m^3^ increase in PM_10_, 10 ppb increase in O_3_, and 1 ppb increase in SO_2_, using different lag structures in Thailand during 1999–2008.

Figure 2 shows the pooled percentage increase (mean and 95% PIs) of mortality associated with air pollutants, using single-pollutant models with different lag structures in Thailand during 1999–2008. All air pollutants had short-term effects on mortality. In general, for all death categories, the effect estimates achieved highest at lag 0 for PM_10_, at lag 0–5 for O_3_, and at lag 0–3 for SO_2_. Thus, we used these lags for each pollutant in the following analyses.

**Figure 3:**
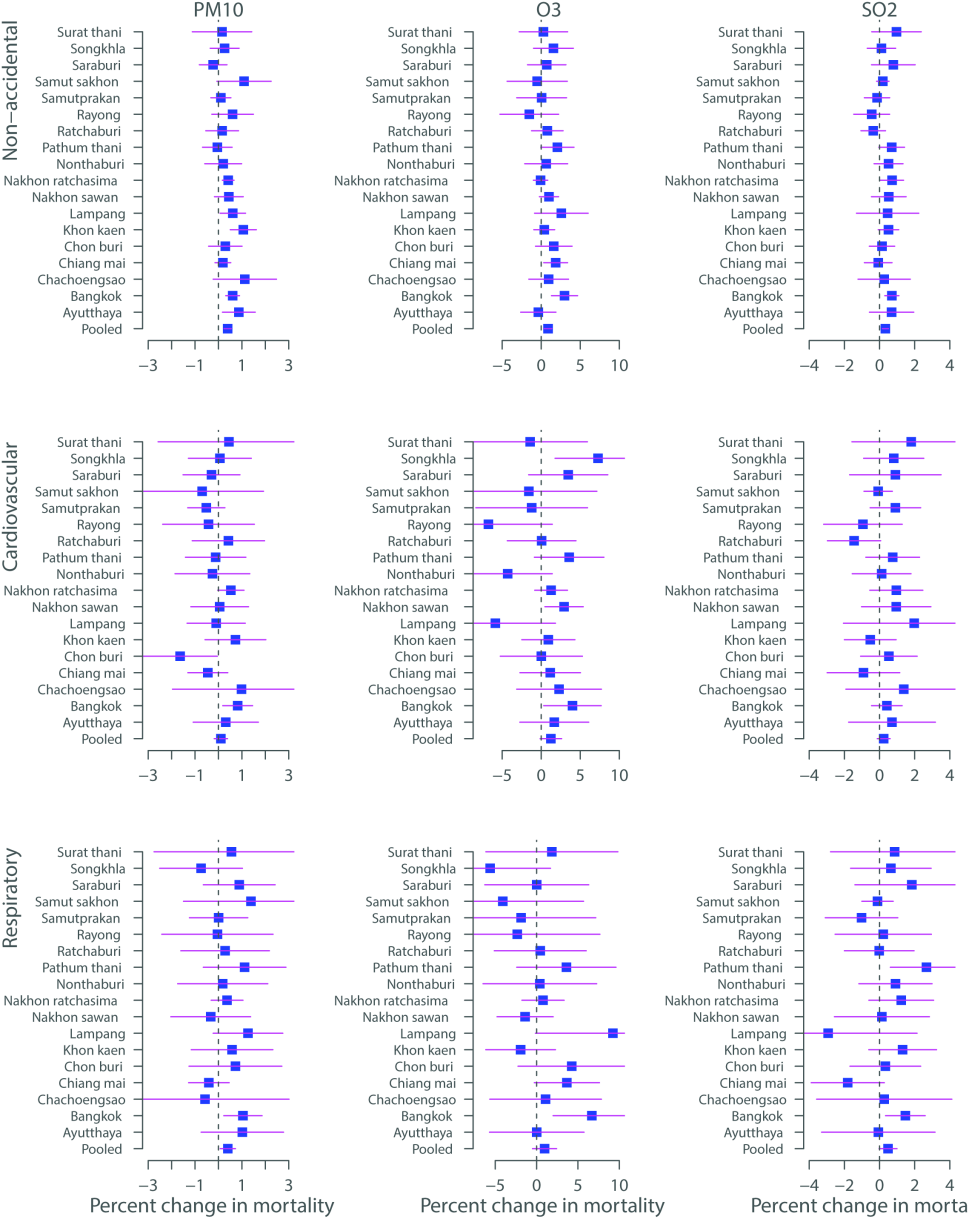
Percentage change (mean and 95% PIs) of mortality associated with a 10 μg/m^3^ increase in PM_10_ (lag 0), 10 ppb increase in O_3_ (lag 0–5), and 1 ppb increase in SO_2_ (lag 0–3) in 18 Thailand provinces during 1999–2008.

Figure 3 shows the percentage increase (mean and 95% PIs) of mortality associated with air pollutants in 18 Thailand provinces during 1999–2008. It is clear that the effect estimates of air pollutants vary greatly by provinces. We observed statistically significant impacts of PM_10_, O_3_ and SO_2_ on non-accidental, cardiovascular, and respiratory mortality in most provinces. Heterogeneity for all associations between air pollutants and mortality was significant (results not shown).

Table 3 shows the pooled percentage increase (mean and 95% PIs) of mortality associated with air pollutants in Thailand during 1999–2008, using single and multi–pollutant models. In the single pollutant models, an increase of 10 μg/m^3^ in PM_10_, 10 ppb in O_3_, 1 ppb in SO_2_ were associated with a 0.40% (95% PI: 0.22, 0.59%), 0.78% (95% PI: 0.20, 1.35%) and 0.34% (95% PI: 0.17, 0.50%) increase of non-accidental mortality, respectively. In the two/three-pollutant models, the estimated associations between air pollutants and mortality were attenuated in comparison with single-pollutant models, but generally the associations between air pollutants and non-accidental mortality remained significant. O_3_ is significantly associated with cardiovascular mortality, while PM_10_ is significantly related to respiratory mortality.

Table 4 presents the pooled effect estimates (mean and 95% PIs) for the increase in mortality associated with air pollutants in three seasons in Thailand during 1999–2008. The associations between air pollutants and mortality differed by season. We observed that the associations between air pollutants and mortality were higher in summer and winter than those in rainy season. For example, an increase of 10 μg/m^3^ in PM_10_ was associated with a 0.51% (95% PI: 0.28, 0.74%), 0.20% (95% PI: −0.09, 0.48%) and 0.35% (95% PI: 0.05, 0.65%) increase of non-accidental mortality in summer, rainy season and winter, respectively.

Sensitivity analyses show that the change of time strata and introducing NO_2_ and CO did not substantially affect the associations of air pollutants with mortality (results not shown), suggesting that our findings are relatively robust in this aspect. However, when we removed temperature and relative humidity from the models, the effect estimates of air pollutants on mortality were higher than those without controlling for temperature and relative humidity (Supplemental Material, Table S2). This suggests that it is necessary to control for temperature and relative humidity when assessing the association between air pollution and mortality. When we used empirical statistical models (generalised linear model and generalised additive model) for this study, the mean effect estimates are similar as those from the Bayesian statistical models. However, the confidence intervals from empirical models are wider than those from Bayesian models.

## Discussion

We used Bayesian statistical inference with the case-crossover design to examine the associations between air pollutants and mortality in 18 provinces in Thailand. To the best of our knowledge, it is the largest study to systematically assess the effects of air pollution on mortality in Thailand. We found that all pollutants (PM_10_, O_3_, and SO_2_) had significant impacts on non-accidental mortality when we used single pollutant models. But the magnitude of effect estimates of air pollutants on mortality were attenuated when two- or three-pollutant models were used. The effects of air pollutants on mortality appeared immediately and lased for 2 days for PM_10_, 5 days for O_3_, and 3 days for SO_2_. The effects of air pollutants on mortality were higher in summer and winter than those in rainy season.

### For PM_10_

In general, our effect estimates associated with PM_10_ in Thailand are consistent with previous studies, except for cardiovascular mortality. For example, our estimate for non-accidental mortality is 0.40% (95% PI, 0.22–0.59%). A study using 75 single-city data worldwide produced an effect estimate of 0.6% (95% confidence interval (CI), 0.5–0.7%)^11^. An USA study with 90 cities generated an estimate of 0.2% (95% CI, 0.1–0.4%)^12^, while an European study of 29 cities gave an estimate of 0.6% (95% CI, 0.4–0.7%)^13^. A study in 14 USA cities generated an estimate of 0.35% (95% CI, 0.2–0.5%)^14^. A multi-city study in four Asian cities (Bangkok, Seoul, Inchon, and Hong Kong) gave a pooled estimate of 0.55% (95% CI, 0.26–0.85%)^10^.

However, some higher estimates have been reported in other developing countries, for example, a Mexico City study showed that an effect estimate of 1.8% (95% CI, 0.9–2.7%), while a result for Santiago, Chile was 1.1% (95% CI, 0.9–1.4%)^15^,^16^. A previous study conducted in Bangkok using data from 1999–2003 produced an estimate of 1.3% (95% CI, 0.8 to 1.7%), which was higher than our result in Bangkok. The reasons might be that previous studies in Bangkok and other developing counties used relatively old data. In addition, it may be that people have recently changed their behaviour to protect themselves from serious air pollution, for example, using mouth mask during the heavily polluted days.

In this study, we found that the estimated effects of PM_10_ on non-accidental mortality were similar to respiratory mortality and were higher than for cardiovascular mortality. This is contrary to our hypothesis that those with cardiopulmonary disease should be more sensitive to air pollution than non-accidental mortality. Many previous studies have confirmed that mortality risks from PM_10_ were higher for cardiovascular and respiratory mortality^10^,^17^. The reason might be that the effects of PM_10_ on mortality varied greatly by province (Figure 3), which makes the pooled effect non-significant for cardiovascular mortality. In addition, the low effect values estimated by our model could be due to publication bias in other single-city studies that are incorporated into the meta-analyses. Because we used the same statistical approach to the 18 large Thailand provinces, our results are free from publication bias. However, the results in Bangkok were similar to previous studies which reported that PM_10_ had significant impacts on cardiovascular and respiratory mortality^2^,^10^. Further detailed studies are needed to explore the reason.

### For O_3_

Our results indicate that O_3_ has a substantial health burden on mortality in Thailand. According to our pooled national estimate from the single pollutant model, a 10 ppb increase in daily O_3_ was associated with an increase of 0.78% (95% PI: 0.20, 1.35%) in non-accidental mortality. The effect estimates for cardiovascular and respiratory mortality were slightly higher than the one for non-accidental mortality. Our results are consistent with previous studies^18^. Meta-analysis study showed that the pooled estimates expressed as the percentage increase in mortality for a 10 ppb increase in daily O_3_ were 0.87% (95% CI, 0.55%–1.18%) for non-accidental mortality, 1.11% (95% CI, 0.68%–1.53%) for cardiovascular mortality; and 0.47% (95% CI, −0.51%–1.47%) for respiratory mortality^19^.

Ozone pollution is still a problem in both urban and rural areas in Thailand^20^, and is related to health problems as we found in this study. In urban areas, its rise was caused primarily by increased usage of motor vehicles, wherein vehicle emissions are a major source of precursor hydrocarbons and nitrogen oxides. In rural areas, agricultural burning and forest fires have contributed to high levels of air pollution, which have increased to critical levels since 2006^1^,^4^. This increased biomass burning could potentially enhance tropical tropospheric ozone. At the same time, the higher UV intensities combined with higher humidity in Thailand's tropical atmosphere result in an increased amount of O_3_^3^. Our results, combined with previous epidemiologic and toxicological evidence on ozone toxicity, suggest that this widespread O_3_ pollution may adversely impact human health.

### For SO_2_

This study showed evidence that outdoor SO_2_ was related to the increased risk of non-accidental mortality in Thailand. The effect estimates of SO_2_ on cardiovascular and respiratory mortality were positive but non-significant. Our findings were generally comparable with most previous studies. Our results in Bangkok were similar to a previous study^21^. A meta-analysis study reported that the excess non-accidental mortality risk associated with a 10 μg/m^3^ increase of SO_2_ was 1.00% (95% CI, 0.75 to 1.24%) in four Asian cities (Bangkok, Hongkong, Shanghai and Wuhan)^21^. Another meta-analysis indicated that a 10 μg/m^3^ increase of SO_2_ was associated with a 0.75% (95% PI, 0.47 to 1.02) increase of non-accidental mortality in 17 Chinese cities^22^. However, some North American studies did not report a significant association between SO_2_ and mortality, which is possibly because of the very low levels of SO_2_ air pollution^23^,^24^.

The estimated mortality risks associated with SO_2_ in Thailand provinces were consistent with previous studies worldwide. However, the burden of disease from exposure to SO_2_ might be lower than that in other countries because the SO_2_ levels in Thailand are much lower than those reported in other developing countries for example, China^21^,^22^. Also, the population in Thailand is lower than in China. However, ambient SO_2_ may still represent a major public health concern in Thailand, as we found it has a significant effect on non-accidental mortality. These findings contribute to the scientific literature on health effects of SO_2_ for low exposure settings typical in developing countries.

### Lagged effects

Understanding the short-term lag pattern of the association between air pollution exposure and mortality is very important for healthcare providers and public health authorities in development of response plans. This study showed that the impacts of air pollution on mortality risks were mainly limited to recent days of exposure (lag 0–5 days) in Thailand. Our findings are consistent with previous studies of air pollution–mortality relationships^22^,^25^, which suggest that the effects of air pollution peak quickly. Therefore, applying timely preventive measures are effective in reducing the health effects of air pollution in Thailand, when extreme air pollution days appear. However, this will depend on timely responses by the health alert system, and better awareness of the dangers of air pollution by government, public health professionals, medical professionals and the general public.

### Multi-pollutant models

We found all pollutants (PM_10_, O_3_ and SO_2_) have a great impact on mortality risks (except PM_10_ and cardiovascular mortality) in Thailand, when we used single pollutant models. Therefore, strict standards for air pollutants should be considered when establishing Thailand's air-quality strategies. When we used the multi-pollutant models, the magnitude of effect estimates of air pollutants were attenuated. These findings were consistent with previous studies^17^,^22^,^25^. This might be caused by the co-linearity between air pollutants, but it is unlikely from the viewpoint of biological mechanisms. However, it is very difficult to separate the effects in multiple pollutants models. Our results of two or three pollutant models should be interpreted with caution, because both SO_2_ and O_3_ are precursors of secondary particles. Thus, our ability to determine precisely the independent effects of individual pollutants on the risk of death is limited.

### Seasonal modification

We found strong evidence that PM_10_, O_3_ and SO_2_ had significant effects on mortality in Thailand. In general, these estimated effects were higher in summer and winter than those in rainy season. Similar findings have been reported by previous studies^26^,^27^. The main reason is that in the rainy season frequent rainfall reduces air pollution (Supplemental Material, Table S3–S5). In addition, in the winter and summer, the effects of air pollution on mortality might be enhanced by both cold and hot temperatures^28^,^29^.

Our study has some limitations. We used ambient pollutant concentrations as individual exposure, which might induce unavoidable measurement error. The local air monitoring stations are located in urban areas at least 50 meters distant from a main road. This may introduce measurement bias to rural areas. Because the monitoring stations are located beside the main roads, it is potential to introduce biases, for example, upwind/downwind of major point sources or highways would influence the individual exposure. The data are at province level, so the effects of air pollutants on mortality in Thailand may be underestimated.

In conclusion, there is clear evidence that air pollution can affect existing health conditions and greatly increase mortality^10^. In addition, air pollution shortens life expectancy and health expectancy^30^,^31^. Thus, it is necessary to pay attention to the dangers of air pollution and to inform the public on how to minimise their risks from serious air pollution. This study highlights the effects of exposure to air pollution on mortality risks in Thailand. Our findings support the Thai government efforts in reducing high levels of air pollution in Thailand, in order to protect the health of the population.

## Methods

### Data collection

This study includes 18 Thailand provinces: Ayutthaya, Bangkok, Chachoengsao, Chiang mai, Chon buri, Khon kaen, Lampang, Nakhon sawan, Nakhon ratchasima, Nonthaburi, Pathum thani, Ratchaburi, Rayong, Samutprakan, Samut sakhon, Saraburi, Songkhla, Surat thani (Figure 1). Our study areas include both urban and sub-urban areas of these provinces, because both are experiencing the same air pollution issues in Thailand and Thai provinces are similar in area to big cities in the USA or China. In addition, death counts, and therefore statistical power, are limited if we restrict the study to urban areas. Previous studies have used similar data to examine the effects of air pollution on mortality in China^32^.

We obtained daily counts of non-accidental death in 18 Thailand provinces from the Ministry of Public Health, Thailand during 01/011999 to 31/12/2008. Causes of death were coded according to the *International Classification of Diseases*, Tenth Revision (ICD-10). We classified the mortality data as deaths due to non-accidental causes (ICD-10: A00–R99), cardiovascular disease (ICD-10: I00–I99), and respiratory disease (ICD-10: J00–J99).

We obtained daily data on air pollution from the Pollution Control Department, Ministry of Natural Resources and Environment for 18 provinces during 1999–2008 (Air Quality and Noise Management Bureau 2010). Monitoring air pollution data started from 2004 for Chachoengsao, and from 2000 for Nakhon sawan. For each province and air pollutant, daily concentration was averaged by fixed air quality monitoring stations within the province. If monitored data for an individual pollutant were insufficient to calculate a daily average, all measurements from that day were excluded for that pollutant and monitor. The averages of daily mean concentrations for five air pollutants were obtained in this study: 24-h average of PM_10_, 24-h average of ozone (O_3_), 24-h average of SO_2_, 24-h average of nitrogen dioxide (NO_2_), and 8-h average of carbon monoxide (CO). We only examined the effects of PM_10_, O_3_ and SO_2_ on mortality in this study, because our preliminary analyses show that NO_2_ and CO did not have significant effects on mortality.

We obtained daily data on weather conditions from the Meteorological Department, Ministry of Information and Communication Technology in 18 provinces from 1999 to 2008 (Meteorological Development Bureau 2010). Daily weather data include four weather variables: mean, minimum, and maximum temperatures (°C), and relative humidity (%).

### Statistical analysis

We used two-stage Bayesian hierarchical statistical models to estimate province-specific and national average associations between daily air pollutants and mortality. In the first stage, we applied a time-stratified case-crossover design, commonly used to examine the effects of short-term exposures, to assess the associations between air pollutants and mortality in each province. The case-crossover design is a form of matched case-control analysis, with each case acting as its own control^33^. This design can successfully control for potential time-invariant confounding factors (e.g., smoking and obesity). When the case-crossover design is applied to time series data, it controls for seasonal and secular trends by matching case and control days in relatively small time windows (e.g., calendar month). This controls for season using a step function rather than a smooth spline function^34^. The case-crossover design fitted by conditional logistic regression (survival regression) is a special case of Poisson regression models^35^. This equivalence provides computational convenience and permits model checking for the case-crossover design using standard log-linear model diagnostics.

In this study, we used a Bayesian statistical inference with Poisson regression model to fit a case-crossover design. It has been well acknowledged that Bayesian methodology provides enormous advantages over traditional methods for epidemiological analyses^36^. We used control days on the same day of the week in the same calendar month as the case day, to adjust for day of the week and to avoid overlap bias^37^. To completely control for the potential effects of ambient temperature on mortality, we included 15-day moving average temperature and 15-day moving average relative humidity with a nonparametric smooth to the models^34^,^38^,^39^.

After establishing the basic model, we introduced the air pollutant concentrations into the models. We examined the associations with different lag structures, including single-day lag (from lag 0 to lag 5) and moving average lag (lag0–1 to lag 0–5). For single-day lag models, a lag of 0 days (lag 0) corresponds to the current-day air pollution concentration, and a lag of 1 day (lag 1) refers to the previous day's concentration; For moving average lag models, lag 0–1 corresponds to an 2-day moving average of air pollution concentration of the current and previous 1 days. For each pollutant, we fitted both single-pollutant and multiple-pollutant models to assess the stability of the associations. The lags producing the highest effect estimates were put into the multi-pollutant models. We used PM_10_ as an example, in the single-pollutant models, PM_10_ was included alone in the model; in the 2-pollutant models, PM_10_ (lag 0) and O_3_ (lag 0–5) (or SO_2_ (lag 0–3)) were included jointly at the same lag. In 3-pollutant models, PM_10_ (lag 0) and O_3_ (lag 0–5) and SO_2_ (lag 0–3)were included jointly at the same lag. To examine whether effects of air pollutants on mortality differed by season, we also stratified analyses by three seasons (summer, rainy season and winter) in Thailand.

In the second stage, we used Bayesian hierarchical meta-analysis to pool the national effect estimates of the association of air pollutants and mortality^40^. This method has been widely used in the area of air pollution and mortality/morbidity^41^. This approach provides a flexible tool to pool risk estimates while accounting for between-province variability (heterogeneity) and within-province statistical error of “true” risks. This Bayesian model produced a posterior probability distribution of pooled estimates, from which we reported the posterior mean and 95% posterior interval (PI).

We conducted a series of sensitivity analyses to assess the robustness of our results by removing temperature and relative humidity, changing time windows (14, 21 and 28 days), introducing NO_2_ and CO into the models. We also used empirical models (generalised linear model and generalised additive models) to check our results.

R software (version 2.30.1) was used to perform all analyses. The “INLA” package was used to fit Bayesian statistical inference^42^ and *TLNISE* package was used to fit Bayesian meta-analysis. The results are presented as the percentage change in daily mortality per 10 μg/m^3^ increase of PM_10_, per 10 ppb increase of O_3_, and per 1 ppb increase of SO_2_.

## Author Contributions

Y.G. conceived and designed this study, and directed its implementation including data analysis, and writing the paper. S.L., B.T. and K.P. prepared the database and reviewed the paper. J.J. and G.W. conducted the quality assurance, reviewed and edited the paper.

## Supplementary Material

Supplementary Information Supplemental information

## Figures and Tables

**Table 1:** Descriptive summary for the study period, daily deaths, air pollutants and weather conditions in 18 Thailand provinces, during 1999–2008

		Mean (Standard deviation)							
Province	Period	Non–accidental death	Cardiovascular death	Respiratory death	PM_10_ (μg/m^3^)	O_3_ (ppb)	SO_2_ (ppb)	Mean temperature (°C)	Relative Humidity (%)
Ayutthaya	1999–2008	7 (2.9)	2 (1.4)	1 (1.1)	61.9 (33.3)	23.2 (9.1)	2.7 (1.6)	28.3 (2.1)	71.9 (9.5)
Bangkok	1999–2008	66 (10.9)	14 (4.1)	8 (3.1)	58.8 (20.6)	13.7 (5.9)	5.7 (1.8)	29.3 (1.7)	70.4 (7.6)
Chachoengsao	2004–2008	6 (2.4)	1 (1.1)	1 (0.9)	42.0 (26.2)	25.6 (8.5)	2.5 (1.6)	25.8 (3.0)	74.3 (9.6)
Chiang mai	1999–2008	21 (5.4)	3 (1.9)	3 (1.8)	53.3 (37.0)	17.5 (8.4)	1.6 (1.3)	26.4 (2.6)	71.6 (9.6)
Chon buri	1999–2008	13 (4.0)	2 (1.6)	2 (1.3)	43.9 (18.9)	17.9 (9.4)	3.9 (2.0)	28.4 (1.4)	74.8 (7.9)
Khon kaen	1999–2008	16 (4.7)	3 (1.7)	2 (1.3)	39.7 (24.8)	16.5 (7.5)	2.7 (2.1)	27.2 (2.8)	72.0 (9.1)
Lampang	1999–2008	10 (3.5)	2 (1.4)	1 (1.2)	53.0 (39.1)	15.3 (9.8)	1.1 (0.9)	28.0 (2.3)	77.6 (8.8)
Nakhon sawan	2000–2008	11 (3.6)	3 (1.7)	1 (1.2)	50.5 (24.5)	25.3 (10.5)	1.9 (1.5)	28.6 (2.2)	70.8 (8.6)
Nakhon ratchasima	1999–2008	21 (5.8)	4 (2.2)	3 (1.7)	58.6 (33.8)	19.9 (8.8)	2.3 (1.7)	27.2 (2.4)	71.6 (8.1)
Nonthaburi	1999–2008	9 (3.1)	2 (1.4)	1 (1.2)	48.7 (23.1)	17.9 (7.7)	4.9 (2.2)	27.8 (2.4)	73.6 (9.7)
Pathum thani	1999–2008	6 (2.9)	1 (1.2)	1 (0.9)	50.1 (26.2)	19.4 (8.8)	4.7 (2.7)	26.9 (3.0)	74.3 (10.4)
Ratchaburi	1999–2008	8 (3.0)	2 (1.3)	1 (1.0)	48.4 (28.0)	21.1 (10.0)	3.7 (2.5)	26.8 (2.8)	72.1 (10.4)
Rayong	1999–2008	5 (2.3)	1 (1.0)	1 (0.8)	42.4 (21.5)	17.9 (9.1)	4.2 (2.0)	25.8 (2.5)	75.0 (10.2)
Samutprakan	1999–2008	8 (3.2)	2 (1.5)	1 (1.0)	94.9 (43.1)	18.5 (8.8)	6.5 (3.3)	28.3 (1.5)	71.3 (7.6)
Samut sakhon	1999–2008	4 (2.1)	1 (0.9)	1 (0.8)	48.5 (23.5)	15.5 (8.7)	13.9 (8.0)	28.2 (1.8)	76.4 (6.6)
Saraburi	1999–2008	6 (2.8)	1 (1.2)	1 (1.0)	62.6 (32.9)	18.3 (9.2)	3 (1.8)	27.9 (2.4)	75.2 (6.8)
Songkhla	1999–2008	9 (3.2)	2 (1.5)	1 (1.1)	38.9 (15.9)	9.9 (7.4)	2.3 (2.0)	27.9 (1.2)	79.7 (5.0)
Surat thani	1999–2008	6 (2.8)	1 (1.1)	1 (0.9)	31.2 (13.5)	12.7 (7.2)	2.3 (1.6)	27.4 (2.8)	70.8 (10.1)

**Table 2:** Average values for Spearman correlation between air pollutants and weather conditions in 18 Thailand provinces

Pollutant	O_3_	SO_2_	Mean temperature	Relative humidity
PM_10_	0.45	0.18	−0.17	−0.39
O_3_		0.03	−0.23	−0.52
SO_2_			0.01	−0.04
Mean temperature				0.03

**Table 3:** Pooled effect estimates (mean and 95% PIs) for the increase in mortality associated with an increase of 10 μg/m^3^ in PM_10_, 10 ppb in O_3_, 1 ppb in SO_2_ in Thailand during 1999–2008, using single–/multi–pollutant models

	% increase (PIs)		
Model	Non–accidental	Cardiovascular	Respiratory
PM_10_	0.40 (0.22, 0.59)	0.13 (−0.16, 0.41)	0.40 (0.07, 0.73)
Adjust for O_3_	0.37 (0.18, 0.56)	0.09 (−0.18, 0.37)	0.32 (−0.02, 0.66)
Adjust for SO_2_	0.37 (0.20, 0.55)	0.09 (−0.19, 0.38)	0.34 (0.01, 0.68)
Adjust for O_3_ and SO_2_	0.35 (0.17, 0.52)	0.07 (−0.21, 0.34)	0.28 (−0.06, 0.62)
O_3_	0.78 (0.20, 1.35)	1.25 (0.15, 2.36)	0.93 (−0.65, 2.51)
Adjust for PM_10_	0.49 (−0.05, 1.03)	1.20 (0.16, 2.25)	0.66 (−0.72, 2.04)
Adjust for SO_2_	0.64 (0.05, 1.23)	1.12 (−0.03, 2.27)	0.69 (−0.85, 2.23)
Adjust for PM_10_ and SO_2_	0.40 (−0.16, 0.96)	1.09 (−0.01, 2.19)	0.47 (−0.91, 1.86)
SO_2_	0.34 (0.17, 0.50)	0.33 (−0.04, 0.70)	0.49 (−0.01, 0.98)
Adjust for PM_10_	0.21 (0.08, 0.41)	0.30 (−0.07, 0.67)	0.38 (−0.09, 0.86)
Adjust for O_3_	0.31 (0.14, 0.47)	0.27 (−0.10, 0.64)	0.44 (−0.05, 0.93)
Adjust for PM_10_ and O_3_	0.23 (0.06, 0.40)	0.26 (−0.12,0.64)	0.36 (−0.11, 0.83)

**Table 4:** Pooled effect estimates (mean and 95% PIs) for the increase in mortality associated with an increase of 10 μg/m^3^ in PM_10_, 10 ppb in O_3_, 1 ppb in SO_2_ in three seasons in 18 Thailand provinces during 1999–2008, with single-pollutant model

		% increase (PIs)		
Pollutant	Season	Non–accidental	Cardiovascular	Respiratory
PM_10_	Summer	0.51 (0.28, 0.74)	0.54 (0.03, 1.05)	0.58 (−0.05, 1.20)
	Rainy	0.20 (−0.09, 0.48)	−0.51 (−1.4, 0.38)	0.24 (−0.56, 1.04)
	Winter	0.35 (0.05, 0.65)	−0.02 (−0.56, 0.51)	0.33 (−0.12, 0.78)
O_3_	Summer	0.52 (−0.15, 1.20)	1.92 (0.39, 3.45)	1.52 (−0.34, 3.38)
	Rainy	0.37 (−0.73, 1.46)	0.37 (−2.10, 2.84)	1.21 (−2.98, 5.40)
	Winter	1.23 (0.27, 2.19)	1.56 (−0.11, 3.24)	0.79 (−1.96, 3.55)
SO_2_	Summer	0.32 (0.02, 0.62)	0.71 (0.007, 1.40)	0.48 (−0.33, 1.30)
	Rainy	0.08 (−0.25, 0.41)	0.15 (−0.60, 0.91)	0.22 (−0.94, 1.38)
	Winter	0.57 (0.31, 0.84)	0.31 (−0.048, 0.87)	0.72 (−0.14, 1.58)

## References

[b1] Vichit-Vadakan N. & Vajanapoom N. Health impact from air pollution in Thailand: current and future challenges. Environ Health Perspect 119, A197–198 (2011). 21531656 10.1289/ehp.1103728 PMC3094431

[b2] Vichit-Vadakan N., Vajanapoom N. & Ostro B. The Public Health and Air Pollution in Asia (PAPA) project: Estimating the mortality effects of particulate matter in Bangkok, Thailand. Environ Health Perspect 116, 1179–1182 (2008). 18795160 10.1289/ehp.10849 PMC2535619

[b3] Suthawaree J. *et al.* Identification of volatile organic compounds in suburban Bangkok, Thailand and their potential for ozone formation. Atmos Res 104, 245–254 (2012).

[b4] Chantara S., Sillapapiromsuk S. & Wiriya W. Atmospheric pollutants in Chiang Mai (Thailand) over a five-year period (2005-2009), their possible sources and relation to air mass movement. Atmos Environ 60, 88–98 (2012).

[b5] Dominici F., Sheppard L. & Clyde M. Health effects of air pollution: A statistical review. Int Stat Rev 71, 243–276 (2003).

[b6] Zanobetti A. *et al.* The temporal pattern of respiratory and heart disease mortality in response to air pollution. Environ Health Perspect 111, 1188 (2003). 12842772 10.1289/ehp.5712 PMC1241573

[b7] Guo Y. *et al.* The short-term effect of air pollution on cardiovascular mortality in Tianjin, China: Comparison of time series and case–crossover analyses. Sci Total Environ 409, 300–306 (2010). 21055792 10.1016/j.scitotenv.2010.10.013

[b8] Guo Y. *et al.* The burden of air pollution on years of life lost in Beijing, China, 2004–08: retrospective regression analysis of daily deaths. BMJ 347, f7139 (2013). 24322399 10.1136/bmj.f7139 PMC3898659

[b9] Guo Y. *et al.* Gaseous air pollution and emergency hospital visits for hypertension in Beijing, China: a time-stratified case-crossover study. Environ Health 9, 57 (2010). 20920362 10.1186/1476-069X-9-57 PMC2972268

[b10] Wong C.-M., Vichit-Vadakan N., Kan H. & Qian Z. Public Health and Air Pollution in Asia (PAPA): a multicity study of short-term effects of air pollution on mortality. Environ Health Perspect 116, 1195 (2008). 18795163 10.1289/ehp.11257 PMC2535622

[b11] Anderson H. R., Atkinson R. W., Peacock J. L., Sweeting M. J. & Marston L. Ambient particulate matter and health effects: publication bias in studies of short-term associations. Epidemiol 16, 155–163 (2005). 10.1097/01.ede.0000152528.22746.0f 15703529

[b12] Dominici F., McDermott A., Daniels M., Zeger S. L. & Samet J. M. Revised analyses of the National Morbidity, Mortality, and Air Pollution Study: Mortality among residents of 90 cities. J Toxicol Environ Health A 68, 1071–1092 (2005). 16024489 10.1080/15287390590935932

[b13] Katsouyanni K. *et al.* Sensitivity analysis of various models of short-term effects of ambient particles on total mortality in 29 cities in APHEA2. Revised Analyses of Time-Series Studies of Air Pollution and Health 157–164 (2003).

[b14] Schwartz J. The effects of particulate air pollution on daily deaths: a multi-city case crossover analysis. Occup Environ Med 61, 956–961 (2004). 15550600 10.1136/oem.2003.008250 PMC1740692

[b15] Castillejos M., Borja-Aburto V. H., Dockery D. W., Gold D. R. & Loomis D. Airborne coarse particles and mortality. Inhal Toxicol 12, 61–72 (2000).

[b16] Ostro B., Sanchez J. M., Aranda C. & Eskeland G. S. Air pollution and mortality: Results from a study of Santiago, Chile. J Expo Anal Env Epid 6, 97–114 (1996). 8777376

[b17] Chen R. *et al.* Association of Particulate Air Pollution With Daily Mortality The China Air Pollution and Health Effects Study. Am J Epidemiol 175, 1173–1181 (2012). 22510278 10.1093/aje/kwr425

[b18] Bell M. L., McDermott A., Zeger S. L., Samet J. M. & Dominici F. Ozone and short-term mortality in 95 US urban communities, 1987–2000. JAMA 292, 2372–2378 (2004). 15547165 10.1001/jama.292.19.2372 PMC3546819

[b19] Bell M. L., Dominici F. & Samet J. M. A meta-analysis of time-series studies of ozone and mortality with comparison to the national morbidity, mortality, and air pollution study. Epidemiol 16, 436–445 (2005). 10.1097/01.ede.0000165817.40152.85 PMC3581312 15951661

[b20] Pochanart P. *et al.* Tropical tropospheric ozone observed in Thailand. Atmos Environ 35, 2657–2668 (2001).

[b21] Kan H. D., Wong C. M., Vichit-Vadakan N., Qian Z. M. & Teams P. P. Short-term association between sulfur dioxide and daily mortality: The Public Health and Air Pollution in Asia (PAPA) study. Environ Res 110, 258–264 (2010). 20122685 10.1016/j.envres.2010.01.006 PMC3392899

[b22] Chen R. J. *et al.* Short-term exposure to sulfur dioxide and daily mortality in 17 Chinese cities: The China air pollution and health effects study (CAPES). Environ Res 118, 101–106, 10.1016/j.envres.2012.07.003 (2012). 22831556 10.1016/j.envres.2012.07.003

[b23] Burnett R. T. *et al.* Association between particulate- and gas-phase components of urban air pollution and daily mortality in eight Canadian cities. Inhal Toxicol 12, 15–39 (2000). 12881885 10.1080/08958370050164851

[b24] Dominici F., Peng R. D., Zeger S. L., White R. H. & Samet J. M. Particulate air pollution and mortality in the United States: Did the risks change from 1987 to 2000? Am J Epidemiol 166, 880–888 (2007). 17728271 10.1093/aje/kwm222

[b25] Guo Y., Jia Y., Pan X., Liu L. & Wichmann H. The association between fine particulate air pollution and hospital emergency room visits for cardiovascular diseases in Beijing, China. Sci Total Environ 407, 4826–4830 (2009). 19501385 10.1016/j.scitotenv.2009.05.022

[b26] Samoli E., Nastos P. T., Paliatsos A. G., Katsouyanni K. & Priftis K. N. Acute effects of air pollution on pediatric asthma exacerbation: Evidence of association and effect modification. Environ Res 111, 418–424 (2011). 21296347 10.1016/j.envres.2011.01.014

[b27] Wong C. M., Ma S., Hedley A. J. & Lam T. H. Effect of air pollution on daily mortality in Hong Kong. Environ Health Perspect 109, 335–340 (2001). 11335180 10.1289/ehp.01109335 PMC1240272

[b28] Stafoggia M., Schwartz J., Forastiere F., Perucci C. A. & Group S. Does temperature modify the association between air pollution and mortality? A multicity case-crossover analysis in Italy. Am J Epidemiol 167, 1476–1485 (2008). 18408228 10.1093/aje/kwn074

[b29] Ren C., Williams G. M., Mengersen K., Morawska L. & Tong S. Does temperature modify short-term effects of ozone on total mortality in 60 large eastern US communities? An assessment using the NMMAPS data. Environ Int 34, 451–458 (2008). 17997483 10.1016/j.envint.2007.10.001

[b30] Wen M. & Gu D. Air Pollution Shortens Life Expectancy and Health Expectancy for Older Adults: The Case of China. J Gerontol A Biol Sci Med Sci (2012). 10.1093/gerona/gls094 22518820

[b31] Pope III C. A., Ezzati M. & Dockery D. W. Fine-particulate air pollution and life expectancy in the United States. N Engl J Med 360, 376–386 (2009). 19164188 10.1056/NEJMsa0805646 PMC3382057

[b32] Tao Y. *et al.* Estimated acute effects of ambient ozone and nitrogen dioxide on mortality in the Pearl River Delta of southern China. Environ Health Perspect 120, 393–398 (2012). 22157208 10.1289/ehp.1103715 PMC3295344

[b33] Maclure M. The case-crossover design: a method for studying transient effects on the risk of acute events. Am J Epidemiol 133, 144–153 (1991). 1985444 10.1093/oxfordjournals.aje.a115853

[b34] Guo Y., Barnett A. G., Pan X., Yu W. & Tong S. The impact of temperature on mortality in Tianjin, China: a case-crossover design with a distributed lag nonlinear model. Environ Health Perspect 119, 1719–1725 (2011). 21827978 10.1289/ehp.1103598 PMC3261984

[b35] Lu Y. & Zeger S. L. On the equivalence of case-crossover and time series methods in environmental epidemiology. Biostat 8, 337–344 (2007). 10.1093/biostatistics/kxl013 16809430

[b36] Dunson D. B. Commentary: practical advantages of Bayesian analysis of epidemiologic data. Am J Epidemiol 153, 1222–1226 (2001). 11415958 10.1093/aje/153.12.1222

[b37] Janes H., Sheppard L. & Lumley T. Overlap bias in the case-crossover design, with application to air pollution exposures. Stat Med 24, 285–300 (2005). 15546133 10.1002/sim.1889

[b38] Guo Y., Punnasiri K., Tong S., Aydin D. & Feychting M. Effects of temperature on mortality in Chiang Mai city, Thailand: a time series study. Environ Health 11, 36 (2012). 22613086 10.1186/1476-069X-11-36 PMC3391976

[b39] Guo Y. *et al.* Extremely cold and hot temperatures increase the risk of ischaemic heart disease mortality: epidemiological evidence from China. Heart 99, 195–203 (2013). 23150195 10.1136/heartjnl-2012-302518

[b40] Dominici F. *et al.* Fine particulate air pollution and hospital admission for cardiovascular and respiratory diseases. JAMA 295, 1127–1134 (2006). 16522832 10.1001/jama.295.10.1127 PMC3543154

[b41] Peng R. D. *et al.* Emergency admissions for cardiovascular and respiratory diseases and the chemical composition of fine particle air pollution. Environ Health Perspect 117, 957–963 (2009). 19590690 10.1289/ehp.0800185 PMC2702413

[b42] Martins T. G., Simpson D., Lindgren F. & Rue H. Bayesian computing with INLA: new features. Comput Stat & Data Analy 67, 68–83 (2013).

