# Supplementary material for: The association between air pollution and mortality in Thailand
Source: Sci Rep. 2014 Jul 1;4:5509. doi: 10.1038/srep05509 (PMC4076679; doi:10.1038/srep05509)
Supplement: Supplementary Information — Supplemental information [file srep05509-s1.pdf]

# Supplemental Material

# The association between air pollution and mortality in Thailand

Yuming Guo[1,2,3*], Shanshan Li[1], Benjawan Tawatsupa[4], Kornwipa Punnasiri[4], Jouni J.K. Jaakkola[3], Gail Williams[1]

[1] Division of Epidemiology and Biostatistics, School of Population Health, The University of Queensland, Brisbane, Australia;

[2] Shanghai Key Laboratory of Meteorology and Health, Shanghai, China

[3] Center for Environmental and Respiratory Health Research, Institute of Health Sciences, University of Oulu, Oulu, Finland;

[4] Health Impact Assessment Division, Department of Heath, Ministry of Public Heath, Thailand.

**Corresponding author**:

Dr. Yuming Guo, Division of Epidemiology and Biostatistics, School of Population Health, The University of Queensland, Brisbane, QLD 4006, Australia; Telephone: +61 7 3346 5265; Email address: y.guo1@uq.edu.au;

# OUTLINE


| Title | Page |
|---|---|
| **Table S1:** Spearman correlation between air pollutants and weather conditions in 18 provinces in Thailand, during 199–2008 | **3 - 4** |
| **Table S2:** Pooled effect estimates (mean and 95% PIs) for the increase in mortality associated with an increase of 10 μg/$m^3$ in $PM_{10}$, 10 ppb in $O_3$, 1 ppb in $SO_2$ in Thailand during 1999–2008, using single pollutant models without controlling for temperature and relative humidity | **5** |
| **Table S3:** Descriptive summary for the study period, daily deaths, air pollutants and weather conditions in 18 provinces in summer (March–June) in Thailand, during 1999–2008 | **6** |
| **Table S4:** Descriptive summary for the study period, daily deaths, air pollutants and weather conditions in 18 provinces in rainy season (July–October) in Thailand, during 1999–2008 | **7** |
| **Table S5:** Descriptive summary for the study period, daily deaths, air pollutants and weather conditions in 18 provinces in winter (November–February) in Thailand, during 1999–2008 | **8** |

**Table S1:** Spearman correlation between air pollutants and weather conditions in 18 provinces in Thailand, during 199–2008.

| Province | Pollutants | $O_3$ | $SO_2$ | Mean temperature | Relative humidity |
|---|---|---|---|---|---|
| Ayutthaya | $PM_{10}$ | 0.67 | 0.17 | –0.39 | –0.69 |
| | $O_3$ | | 0.15 | –0.17 | –0.7 |
| | $SO_2$ | | | 0.11 | –0.08 |
| | Mean temperature | | | | 0.24 |
| Bangkok | $PM_{10}$ | 0.49 | 0.37 | –0.41 | –0.45 |
| | $O_3$ | | 0.05 | –0.13 | –0.63 |
| | $SO_2$ | | | –0.03 | –0.03 |
| | Mean temperature | | | | 0.12 |
| Chachoengsao | $PM_{10}$ | 0.44 | 0.21 | –0.57 | –0.43 |
| | $O_3$ | | 0.12 | –0.46 | –0.44 |
| | $SO_2$ | | | –0.15 | –0.09 |
| | Mean temperature | | | | 0.06 |
| Chiang mai | $PM_{10}$ | 0.53 | 0.29 | –0.14 | –0.59 |
| | $O_3$ | | 0.08 | 0.08 | –0.75 |
| | $SO_2$ | | | –0.02 | –0.19 |
| | Mean temperature | | | | 0.01 |
| Chon buri | $PM_{10}$ | 0.5 | 0.08 | –0.26 | –0.38 |
| | $O_3$ | | –0.07 | –0.43 | –0.64 |
| | $SO_2$ | | | 0.11 | –0.02 |
| | Mean temperature | | | | 0.2 |
| Khon kaen | $PM_{10}$ | 0.26 | 0.32 | –0.2 | –0.43 |
| | $O_3$ | | –0.02 | 0.12 | –0.59 |
| | $SO_2$ | | | –0.15 | –0.27 |
| | Mean temperature | | | | 0.04 |
| Lampang | $PM_{10}$ | 0.67 | 0.22 | 0.01 | –0.66 |
| | $O_3$ | | 0.14 | 0.16 | –0.7 |
| | $SO_2$ | | | –0.02 | –0.13 |
| | Mean temperature | | | | –0.09 |
| Nakhon sawan | $PM_{10}$ | 0.62 | 0.32 | –0.25 | –0.61 |
| | $O_3$ | | 0.28 | –0.04 | –0.68 |
| | $SO_2$ | | | –0.08 | –0.28 |
| | Mean temperature | | | | 0.04 |
| Nakhon ratchasima | $PM_{10}$ | 0.26 | –0.03 | –0.29 | –0.4 |
| | $O_3$ | | 0.06 | –0.05 | –0.56 |
| | $SO_2$ | | | –0.09 | –0.02 |
| | Mean temperature | | | | 0.15 |
| Nonthaburi | $PM_{10}$ | 0.54 | 0.35 | –0.51 | –0.51 |
| | $O_3$ | | 0.23 | –0.31 | –0.62 |
| | $SO_2$ | | | –0.2 | –0.11 |
| | Mean temperature | | | | 0.20 |

| | | | | | |
|---|---|---|---|---|---|
| Pathum thani | $PM_{10}$ | 0.34 | 0.17 | –0.11 | –0.42 |
| | $O_3$ | | 0.2 | –0.35 | –0.52 |
| | $SO_2$ | | | 0.08 | –0.2 |
| | Mean temperature | | | | 0.16 |
| Ratchaburi | $PM_{10}$ | 0.69 | 0.42 | –0.58 | –0.23 |
| | $O_3$ | | 0.38 | –0.46 | –0.42 |
| | $SO_2$ | | | –0.31 | –0.22 |
| | Mean temperature | | | | –0.16 |
| Rayong | $PM_{10}$ | 0.48 | 0.1 | –0.41 | –0.15 |
| | $O_3$ | | 0.03 | –0.52 | –0.36 |
| | $SO_2$ | | | –0.04 | 0.05 |
| | Mean temperature | | | | –0.18 |
| Samutprakan | $PM_{10}$ | 0.59 | 0.16 | –0.26 | –0.29 |
| | $O_3$ | | 0.05 | –0.38 | –0.51 |
| | $SO_2$ | | | 0.11 | 0.15 |
| | Mean temperature | | | | 0.18 |
| Samut sakhon | $PM_{10}$ | 0.52 | 0.6 | –0.46 | –0.38 |
| | $O_3$ | | 0.27 | –0.43 | –0.37 |
| | $SO_2$ | | | –0.33 | –0.33 |
| | Mean temperature | | | | 0.13 |
| Saraburi | $PM_{10}$ | 0.26 | 0.12 | –0.31 | –0.23 |
| | $O_3$ | | 0.28 | –0.38 | –0.54 |
| | $SO_2$ | | | –0.31 | –0.22 |
| | Mean temperature | | | | –0.07 |
| Songkhla | $PM_{10}$ | 0.13 | 0.08 | 0.17 | –0.16 |
| | $O_3$ | | 0.05 | –0.31 | –0.17 |
| | $SO_2$ | | | 0.09 | –0.03 |
| | Mean temperature | | | | –0.62 |
| Surat thani | $PM_{10}$ | 0.47 | 0.03 | –0.26 | –0.39 |
| | $O_3$ | | –0.03 | –0.49 | –0.50 |
| | $SO_2$ | | | 0.01 | –0.13 |
| | Mean temperature | | | | 0.15 |

**Table S2:** Pooled effect estimates (mean and 95% posterior intervals) for the increase in mortality associated with an increase of 10 μg/m$^3$ in $PM_{10}$, 10 ppb in $O_3$, 1 ppb in $SO_2$ in Thailand during 1999–2008, using single pollutant models without controlling for temperature and relative humidity.

| | % increase (95% posterior intervals) | | |
|---|---|---|---|
| Pollutant | Non–accidental | Cardiovascular | Respiratory |
| $PM_{10}$ | 0.49 (0.3, 0.67) | 0.29 (-0.02, 0.61) | 0.40 (0.06, 0.75) |
| $O_3$ | 1.26 (0.64, 1.89) | 1.88 (0.51, 3.25) | 1.30 (-0.28, 2.87) |
| $SO_2$ | 0.38 (0.20, 0.57) | 0.28 (-0.08, 0.63) | 0.55 (0.06, 1.05) |

**Table S3:** Descriptive summary for the study period, daily deaths, air pollutants and weather conditions in 18 provinces in summer (March–June) in Thailand, during 1999–2008

| Province | Period | Mean (Standard deviation) | | | | | | | |
|---|---|---|---|---|---|---|---|---|---|
| | | Non–accidental | Cardiovascular | Respiratory | $PM_{10}$ | $O_3$ | $SO_2$ | Mean temperature | Relative humidity |
| Ayutthaya | 1999–2008 | 7 (2.9) | 2 (1.3) | 1 (1.1) | 52.5 (23.7) | 25 (8.4) | 2.8 (1.6) | 29.9 (1.4) | 72.4 (8.1) |
| Bangkok | 1999–2008 | 67 (10.6) | 13 (4.1) | 8 (3) | 51.8 (14.3) | 13.6 (5.9) | 5.5 (1.7) | 30.3 (1.4) | 72.2 (6.4) |
| Chachoengsao | 2004–2008 | 6 (2.5) | 1 (1) | 1 (1) | 30.7 (11.9) | 26.2 (6.7) | 1.9 (1.2) | 27.7 (2.1) | 73.1 (10.3) |
| Chiang mai | 1999–2008 | 21 (5.2) | 3 (1.9) | 3 (1.8) | 61.5 (48) | 22.4 (9.7) | 1.6 (1.3) | 28.1 (1.7) | 67.8 (10.7) |
| Chon buri | 1999–2008 | 13 (3.9) | 2 (1.6) | 2 (1.3) | 39.4 (15.5) | 15.7 (6.6) | 3.8 (1.8) | 29.3 (1.1) | 76.3 (5.2) |
| Khon kaen | 1999–2008 | 17 (4.4) | 3 (1.7) | 2 (1.3) | 35.2 (20.6) | 20.7 (8) | 2.3 (1.5) | 29 (2.1) | 70.9 (9.4) |
| Lampang | 1999–2008 | 11 (3.6) | 2 (1.4) | 1 (1.2) | 59.9 (45.8) | 21.1 (10.9) | 1.2 (1.1) | 29.5 (1.8) | 75.4 (10) |
| Nakhon sawan | 2000–2008 | 11 (3.6) | 3 (1.8) | 1 (1.2) | 45.9 (19.9) | 28.8 (10.8) | 1.8 (1.5) | 30.3 (1.8) | 69.9 (8) |
| Nakhon ratchasima | 1999–2008 | 21 (5.5) | 4 (2.2) | 3 (1.7) | 51 (23.7) | 23.8 (9) | 2.1 (1.3) | 28.8 (1.7) | 70.8 (8.1) |
| Nonthaburi | 1999–2008 | 9 (3.2) | 2 (1.5) | 1 (1.3) | 39.7 (14.1) | 18 (7.2) | 4.6 (2) | 29.5 (1.7) | 72.9 (9.5) |
| Pathum thani | 1999–2008 | 6 (2.9) | 1 (1.2) | 1 (0.9) | 53.5 (27.8) | 20.3 (8.6) | 5.4 (3.1) | 28.8 (2.3) | 73.7 (11.2) |
| Ratchaburi | 1999–2008 | 8 (3) | 2 (1.3) | 1 (1) | 36.8 (16.9) | 20.4 (8.7) | 3.1 (2.4) | 28.8 (1.9) | 67.4 (11.8) |
| Rayong | 1999–2008 | 5 (2.3) | 1 (1) | 1 (0.8) | 38.9 (20.7) | 16.5 (6.3) | 4.2 (2.3) | 27.7 (1.8) | 70.8 (12.1) |
| Samutprakan | 1999–2008 | 8 (3.1) | 2 (1.5) | 1 (1) | 83.8 (35.1) | 17.8 (8) | 7.1 (4) | 29.1 (1.1) | 73.3 (5.8) |
| Samut sakhon | 1999–2008 | 4 (2.1) | 1 (0.9) | 1 (0.8) | 38.2 (15) | 14.4 (7.6) | 10.2 (6.2) | 29.2 (1.2) | 77.2 (5.1) |
| Saraburi | 1999–2008 | 6 (2.8) | 1 (1.2) | 1 (1) | 54.8 (21.5) | 19 (8.1) | 2.6 (1.3) | 29.5 (1.7) | 73.5 (7.6) |
| Songkhla | 1999–2008 | 9 (3.1) | 2 (1.5) | 1 (1.1) | 38.5 (13) | 9.1 (5.9) | 2.4 (2.2) | 28.6 (1) | 78.7 (4.2) |
| Surat thani | 1999–2008 | 6 (2.6) | 1 (1.1) | 1 (0.9) | 31.9 (13.4) | 11.8 (5) | 2.4 (1.6) | 29.1 (2.2) | 69.6 (9.4) |

**Table S4:** Descriptive summary for the study period, daily deaths, air pollutants and weather conditions in 18 provinces in rainy season (July–October) in Thailand, during 1999–2008

| Province | Period | Mean (Standard deviation) | | | | | | | |
|---|---|---|---|---|---|---|---|---|---|
| | | Non–accidental | Cardiovascular | Respiratory | $PM_{10}$ | $O_3$ | $SO_2$ | Mean temperature | Relative humidity |
| Ayutthaya | 1999–2008 | 7 (2.8) | 2 (1.3) | 1 (1.1) | 42.3 (21.5) | 16.7 (6.6) | 2.8 (1.9) | 28.5 (1) | 79 (6.1) |
| Bangkok | 1999–2008 | 68 (9.7) | 14 (4) | 9 (3) | 51.3 (13.6) | 10.7 (4.3) | 5.6 (1.5) | 29.4 (1) | 73.5 (6) |
| Chachoengsao | 2004–2008 | 6 (2.5) | 1 (1.1) | 1 (0.9) | 31.1 (16.2) | 21.2 (6.2) | 2.5 (1.4) | 27 (1.3) | 79.8 (7.1) |
| Chiang mai | 1999–2008 | 21 (5.4) | 3 (1.9) | 3 (1.8) | 33.9 (14.4) | 11.7 (3.9) | 1.5 (1.4) | 27.3 (1.1) | 78.7 (4.8) |
| Chon buri | 1999–2008 | 13 (3.8) | 2 (1.7) | 2 (1.2) | 36.5 (11.7) | 12.9 (6) | 4.2 (2.4) | 28.6 (1) | 78.2 (5.7) |
| Khon kaen | 1999–2008 | 17 (4.7) | 3 (1.7) | 2 (1.3) | 30.9 (16) | 12.1 (5.2) | 2.2 (1.5) | 27.8 (1.2) | 78.8 (6.2) |
| Lampang | 1999–2008 | 10 (3.5) | 2 (1.4) | 1 (1.2) | 31.3 (12) | 8.1 (3.1) | 0.9 (0.7) | 28.4 (1) | 83.9 (5) |
| Nakhon sawan | 2000–2008 | 11 (3.6) | 3 (1.7) | 1 (1.2) | 36.9 (15.2) | 16.6 (6.2) | 1.2 (1) | 28.6 (1) | 77.9 (5.4) |
| Nakhon ratchasima | 1999–2008 | 20 (5.9) | 4 (2.1) | 2 (1.7) | 45 (25.4) | 14.4 (5.3) | 2.3 (1.7) | 27.6 (1.2) | 76.8 (6) |
| Nonthaburi | 1999–2008 | 8 (2.9) | 2 (1.4) | 1 (1.2) | 39.4 (12) | 13.2 (4.9) | 4.6 (1.7) | 28.2 (1.1) | 81.5 (5.2) |
| Pathum thani | 1999–2008 | 7 (2.9) | 1 (1.3) | 1 (1) | 38 (18.1) | 14.2 (6.5) | 4 (1.9) | 27.9 (1.4) | 81.7 (7.3) |
| Ratchaburi | 1999–2008 | 8 (2.9) | 2 (1.3) | 1 (1) | 36.2 (18.3) | 14.3 (6.4) | 3 (1.6) | 27.5 (1.2) | 79.8 (5.3) |
| Rayong | 1999–2008 | 5 (2.4) | 1 (1) | 1 (0.8) | 33.9 (15.1) | 12.2 (4.7) | 4.2 (1.9) | 26.2 (1) | 82.5 (4.4) |
| Samutprakan | 1999–2008 | 8 (3.2) | 2 (1.4) | 1 (1) | 77 (29.4) | 13.4 (5.8) | 6.2 (2.5) | 28.6 (1) | 72.7 (5.4) |
| Samut sakhon | 1999–2008 | 4 (2.2) | 1 (1) | 1 (0.8) | 43.6 (16.1) | 10.3 (5.6) | 13.8 (5.7) | 28.8 (1.1) | 78.2 (6.1) |
| Saraburi | 1999–2008 | 6 (2.8) | 1 (1.2) | 1 (0.9) | 52.2 (25.7) | 12 (5.6) | 2.4 (1.3) | 28.4 (1) | 80.5 (4.5) |
| Songkhla | 1999–2008 | 9 (3.3) | 2 (1.4) | 1 (1) | 42.8 (18.5) | 7.2 (4.9) | 2.4 (1.6) | 28.2 (1) | 79.5 (4.9) |
| Surat thani | 1999–2008 | 6 (2.9) | 1 (1) | 1 (0.9) | 24.7 (9.1) | 8.8 (3) | 2.1 (1.5) | 28 (1.3) | 77.9 (8) |

**Table S5:** Descriptive summary for the study period, daily deaths, air pollutants and weather conditions in 18 provinces in winter (November–February) in Thailand, during 1999–2008

| Province | Period | Mean (Standard deviation) | | | | | | | |
|---|---|---|---|---|---|---|---|---|---|
| | | Non–accidental | Cardiovascular | Respiratory | $PM_{10}$ | $O_3$ | $SO_2$ | Mean temperature | Relative humidity |
| Ayutthaya | 1999–2008 | 7 (3.1) | 2 (1.5) | 1 (1.1) | 90 (31.8) | 28.2 (7.9) | 2.5 (1.4) | 26.5 (2.2) | 64.9 (8.2) |
| Bangkok | 1999–2008 | 65 (12.1) | 14 (4.2) | 8 (3.2) | 73.5 (23.9) | 17 (5.5) | 6 (2) | 28 (1.8) | 65.5 (7.7) |
| Chachoengsao | 2004–2008 | 5 (2.2) | 1 (1.1) | 1 (0.8) | 62.6 (30.3) | 29.9 (9.7) | 2.9 (1.9) | 22.8 (2.9) | 69.6 (8.2) |
| Chiang mai | 1999–2008 | 20 (5.5) | 3 (2) | 3 (1.9) | 64.8 (32) | 18.5 (6.8) | 1.8 (1.3) | 23.6 (2.2) | 68.4 (7.8) |
| Chon buri | 1999–2008 | 12 (4.1) | 3 (1.7) | 2 (1.3) | 56.1 (22) | 25.5 (10.1) | 3.6 (1.6) | 27.3 (1.5) | 69.8 (9.5) |
| Khon kaen | 1999–2008 | 16 (5) | 3 (1.8) | 2 (1.3) | 53.3 (29.8) | 16.7 (6.4) | 3.5 (2.8) | 24.7 (2.8) | 66.2 (6.4) |
| Lampang | 1999–2008 | 10 (3.6) | 2 (1.4) | 1 (1.2) | 68 (40.2) | 16.9 (8.2) | 1.2 (0.8) | 26 (2.2) | 73.5 (6.8) |
| Nakhon sawan | 2000–2008 | 11 (3.7) | 3 (1.8) | 1 (1.2) | 67.6 (25.5) | 30.5 (8) | 2.4 (1.6) | 26.9 (2.2) | 65.9 (7.2) |
| Nakhon ratchasima | 1999–2008 | 20 (5.9) | 4 (2.2) | 3 (1.7) | 80.9 (38.8) | 22.1 (8.6) | 2.4 (2) | 25.1 (2.4) | 66.7 (6.4) |
| Nonthaburi | 1999–2008 | 8 (3.3) | 2 (1.5) | 1 (1.2) | 66.6 (27.5) | 22.7 (7.7) | 5.5 (2.6) | 25.8 (2.4) | 66.2 (6.9) |
| Pathum thani | 1999–2008 | 6 (2.9) | 1 (1.2) | 1 (0.9) | 58.8 (27) | 23.9 (8.4) | 4.9 (2.8) | 24.2 (2.8) | 67.7 (7) |
| Ratchaburi | 1999–2008 | 8 (3.2) | 2 (1.4) | 1 (1.1) | 73.2 (29.4) | 28.6 (8.9) | 5 (2.8) | 23.9 (2.5) | 69.1 (7.9) |
| Rayong | 1999–2008 | 5 (2.3) | 1 (1) | 1 (0.8) | 54.1 (22.4) | 24.8 (10) | 4.2 (2) | 23.5 (2.3) | 71.9 (8) |
| Samutprakan | 1999–2008 | 8 (3.3) | 2 (1.4) | 1 (1.1) | 124.8 (46.7) | 24.7 (8.5) | 6.1 (3.3) | 27.2 (1.7) | 67.7 (9.6) |
| Samut sakhon | 1999–2008 | 4 (2.2) | 1 (0.9) | 1 (0.8) | 64 (28.5) | 22 (8.4) | 17.8 (9.7) | 26.6 (1.7) | 73.8 (7.4) |
| Saraburi | 1999–2008 | 6 (2.9) | 1 (1.3) | 1 (1) | 80.9 (40.3) | 24.3 (9.1) | 3.8 (2.2) | 25.8 (2.4) | 72.4 (4.7) |
| Songkhla | 1999–2008 | 9 (3.2) | 2 (1.5) | 1 (1.1) | 34.6 (13.9) | 13.5 (9.1) | 2.1 (2.3) | 27 (0.9) | 80.9 (5.6) |
| Surat thani | 1999–2008 | 6 (2.9) | 1 (1.1) | 1 (1) | 37.7 (14.5) | 17.7 (9.2) | 2.2 (1.6) | 24.7 (2.8) | 62.6 (5.9) |
